# Outcomes of Adult In-Hospital Cardiac Arrest Treated with Targeted Temperature Management: A Retrospective Cohort Study

**DOI:** 10.1371/journal.pone.0166148

**Published:** 2016-11-07

**Authors:** Chih-Hung Wang, Chien-Hua Huang, Wei-Tien Chang, Min-Shan Tsai, Ping-Hsun Yu, Yen-Wen Wu, Wen-Jone Chen

**Affiliations:** 1 Department of Emergency Medicine, National Taiwan University Hospital and National Taiwan University College of Medicine, Taipei, Taiwan; 2 Graduate Institute of Clinical Medicine, College of Medicine, National Taiwan University, Taipei, Taiwan; 3 Department of Emergency Medicine, Taipei Hospital, Ministry of Health and Welfare, New Taipei City, Taiwan; 4 Departments of Internal Medicine and Nuclear Medicine, National Taiwan University Hospital and National Taiwan University College of Medicine, Taipei, Taiwan; 5 Department of Nuclear Medicine and Cardiology Division of Cardiovascular Medical Center, Far Eastern Memorial Hospital, New Taipei City, Taiwan; 6 National Yang-Ming University School of Medicine, Taipei, Taiwan; 7 Department of Emergency Medicine, Lotung Poh-Ai Hospital, Yilan, Taiwan; Azienda Ospedaliero Universitaria Careggi, ITALY

## Abstract

**Aim:**

Targeted temperature management (TTM) for in-hospital cardiac arrest (IHCA) is given different recommendation levels within international resuscitation guidelines. We aimed to identify whether TTM would be associated with favourable outcomes following IHCA and to determine which factors would influence the decision to implement TTM.

**Methods:**

We conducted a retrospective observational study in a single medical centre. We included adult patients suffering IHCA between 2006 and 2014. We used multivariable logistic regression analysis to evaluate associations between independent variables and outcomes.

**Results:**

We included a total of 678 patients in our analysis; only 22 (3.2%) patients received TTM. Most (81.1%) patients met at least one exclusion criteria for TTM. In all, 144 (21.2%) patients survived to hospital discharge; among them, 60 (8.8%) patients displayed favourable neurological status at discharge. TTM use was significantly associated with favourable neurological outcome (OR: 3.74, 95% confidence interval [CI]: 1.19–11.00; *p*-value = 0.02), but it was not associated with survival (OR: 1.41, 95% CI: 0.54–3.66; *p*-value = 0.48). Arrest in the emergency department was positively associated with TTM use (OR: 22.48, 95% CI: 8.40–67.64; *p* value < 0.001) and having vasopressors in place at the time of arrest was inversely associated with TTM use (OR: 0.08, 95% CI: 0.004–0.42; *p*-value = 0.02).

**Conclusion:**

TTM might be associated with favourable neurological outcome of IHCA patients, irrespective of arrest rhythms. The prevalence of proposed exclusion criteria for TTM was high among IHCA patients, but these factors did not influence the use of TTM in clinical practice or neurological outcomes after IHCA.

## Introduction

In the United States, approximately 209,000 patients experience in-hospital cardiac arrest (IHCA) each year [1]. Even if patients initially achieve return of spontaneous circulation (ROSC), many still suffer from post-cardiac arrest syndrome (PCAS), which comprises anoxic brain injury, myocardial dysfunction, and systemic ischemia/reperfusion response [2]. Roughly 20% of IHCA patients survive to hospital discharge, and, among them, approximately 28% suffer from significant neurological disability [3].

In 2002, therapeutic hypothermia, which was defined cooling a patient’s body to 32°C to 34°C, was shown to improve outcomes of out-of-hospital cardiac arrest (OHCA) with shockable rhythms [4,5]. Since 2005, resuscitation guidelines have also recommended hypothermia for IHCA with all types of rhythms [6,7]. After the trial of targeted temperature management (TTM) published in 2013 [8], which found no benefit of cooling to 33°C compared with 36°C, the effectiveness of therapeutic hypothermia has been questioned. Subsequently, temperature management after cardiac arrest is referred to as “TTM” to reflect the fact that a variety of temperature targets are used in clinical practice.

Results of the 2013 TTM trial were interpreted from varied perspectives [9], and, in its 2015 guidelines for IHCA patients, the American Heart Association (AHA) [10] classified TTM as a Class I indication; in contrast, the European Resuscitation Council (ERC) [11] graded TTM as a weak recommendation. Hypothermia has been shown to moderate various destructive mechanisms in PCAS, especially those related to anoxic brain injury [12]. Nonetheless, most IHCA patients died of multiple organ failure rather than brain injury [13]. Kory et al. [14] and Nichol et al. [15] demonstrated that TTM might not improve outcomes of IHCA. The rate of TTM use after IHCA has remained low and, according to a representative nationwide investigation in the United States by Mikkelsen et al. [16], the estimated rate of use of TTM is only about 2%.

Recently, Perman et al. [17] indicated that TTM might be effective for IHCA with non-shockable rhythms. However, restricting TTM use to patients with certain rhythm types limited the extrapolation of their results [17]. Therefore, in this current analysis, we aimed to investigate whether TTM was effective for IHCA. Also, we attempted to determine which factors would be associated with the administration of TTM.

## Materials and Methods

### Setting

We conducted a retrospective cohort study at the National Taiwan University Hospital (NTUH), which is a tertiary care centre. The study was performed in accordance with the amended Declaration of Helsinki. Before data collection, the Research Ethics Committee of the NTUH approved this study and waived the requirement for informed consent (Reference number: 201601045RINB).

### Participants

We screened patients who had suffered IHCA at the NTUH between 2006 and 2014. We included patients who met the following criteria: (1) age 18 years or older; (2) documentation of pulselessness with performance of chest compression for at least 2 minutes; (3) absences of a do-not-resuscitate order; and (4) qualification as a candidate for TTM (i.e., unconsciousness after sustained ROSC). If multiple cardiac arrest events occurred in a single patient, we only recorded the first event of the same hospitalization. We excluded patients who had suffered a cardiac arrest related to major trauma and patients who received extracorporeal cardiopulmonary resuscitation (ECPR).

### Data Collection and Outcome Measures

We recorded the following information for each patient: age, gender, comorbidities, reasons for potential exclusion from TTM, variables derived from the Utstein template [18], critical interventions implemented at the time of cardiac arrest or after sustained ROSC, and vital signs after sustained ROSC.

The primary outcomes were clinical status at hospital discharge, including neurological outcome and survival; the secondary outcome was TTM use. Favourable neurological outcome was defined as a score of 1 or 2 on the Cerebral Performance Category (CPC) scale [19]. Patients with a CPC score of 1 or 2 have sufficient cerebral function to live independently. The research assistants who were blind to the study objective retrospectively determined the CPC score for each patient by reviewing the medical records.

### Protocols for TTM and Withdrawal of Life-sustaining Therapy

At the NTUH, TTM was initiated by infusion of 4°C isotonic saline. A peripheral cooling system device that included either a cooling blanket or the Arctic Sun^®^ cooling system (Medivance, Inc., Louisville, CO, USA) was applied to achieve the targeted temperature of 33°C within 4 to 6 hours after ROSC. An oesophageal temperature probe was used to monitor core body temperature. The targeted temperature was maintained for 24 hours, followed by rewarming at a rate of 0.25°C per hour. A body temperature of 36.5°C was usually achieved 12 to 16 hours after rewarming. Propofol infusion was used for sedation and cisatracurium infusion used for paralysis if shivering occurred. Mean arterial pressure was maintained > 90 mmHg and urine output >30 ml/hr. PaCO_2_ was kept at 35–45 mmHg and PaO_2_ 60–200 mmHg by adjusting the ventilator setting.

Patients were considered eligible for TTM if they remained comatose after sustained ROSC (a score of < 8 on the Glasgow Coma Scale [i.e. patients were not able to obey verbal commands]). Exclusion criteria for TTM included: (1) suspected or documented pregnancy, (2) coagulopathy (prothrombin time > 16 sec, partial thromboplastin time > 40 sec, or international normalized ratio > 1.7) [20], (3) thrombocytopenia (platelet count < 100,000/μL) [21], (4) intracranial haemorrhage, (5) suspected or documented active bleeding (bleeding sites included gastrointestinal tract, genitourinary tract, trachea, extremities, intra-abdomen, and head and neck area), (6) acute stroke, (7) no favourable neurological status within 24 hours before cardiac arrest (a score of 3 or 4 on the CPC scale) [19], and (8) hypotension after sustained ROSC (systolic blood pressure < 80 mm Hg) [8]. In clinical practice, TTM was used, even if patients met anyone of these exclusion criteria, if the risk-benefit profile was deemed acceptable under the clinician’s discretion.

At the NTUH, neuroprognostication was conducted by a daily check of brainstem reflex, as well as electroencephalography on the 3rd and 7th days after ROSC. Before 2015, withdrawal of life-sustaining therapy (WLST) for PCAS patients was not allowed by legislation in Taiwan. Therefore, the results of neuroprognostication may have served only as the basis for surrogates and clinicians to decide to withhold further aggressive life-sustaining therapy.

### Statistical Analysis

We used R 2.15.3 software (R Foundation for Statistical Computing, Vienna, Austria) for data analysis. Categorical data were expressed as counts and proportions; continuous data were expressed as means and standard deviations. Categorical variables were compared by the Fisher’s exact test, and continuous variables were examined by the Wilcoxon rank-sum test. A two-tailed *p*-value of < 0.05 was considered statistically significant.

We conducted multivariable logistic regression analyses to test the associations between independent variables and outcomes. All available independent variables were considered in the regression model, regardless of whether they were significant by univariate analysis. The stepwise variable selection procedure was applied to obtain the final regression model. Significance levels for entry and to stay were set at 0.15 to avoid exclusion of potential candidate variables. The final regression model was identified by excluding individual variables with a *p*-value > 0.05 until all regression coefficients were statistically significant.

For the primary outcome, if TTM was not significantly associated with clinical status at hospital discharge, the variable of TTM would be forcibly entered into the final model to assess its effect estimate, irrespective of its *p*-value; for the secondary outcome, if none of the reasons for potential exclusion significantly correlated with TTM use, the variable with the smallest *p*-value would also be forcibly entered into the final model. We assessed the goodness-of-fit of the fitted regression model using *c* statistics, adjusted generalized *R*^2^, and the Hosmer-Lemeshow goodness-of-fit test.

## Results

**Fig 1:**
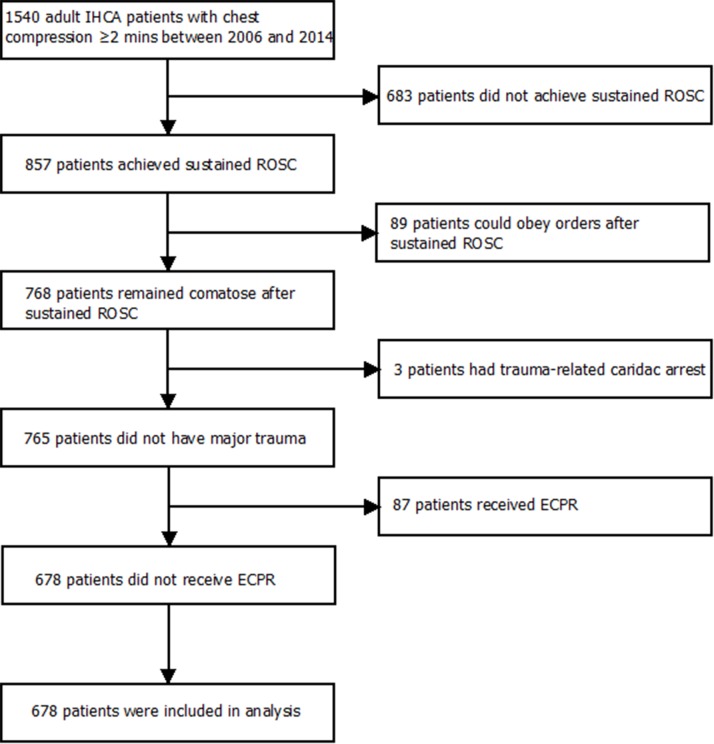
Flow diagram of patient selection. ECPR, extracorporeal cardiopulmonary resuscitation; IHCA, in-hospital cardiac arrest; ROSC, return of spontaneous circulation

We identified a total of 678 patients who met the inclusion criteria and were not excluded from the final analysis (Fig 1). Of these patients, 22 (3.2%) received TTM. The baseline characteristics, peri-arrest events, and outcomes of the included patients were presented in Tables 1–3. All variables listed in Tables 1 to 3, except post-ROSC body temperature, were included in the variable selection procedure for the primary outcome. As shown in Table 4, TTM was significantly associated with favourable neurological outcome (odds ratio [OR]: 3.74, 95% confidence interval [CI]: 1.19–11.00; *p*-value = 0.02), but it was not associated with survival to hospital discharge (OR: 1.41, 95% CI: 0.54–3.66; *p*-value = 0.48).

**Table 1:** Baseline characteristics of study patients.

Variables	All patients (n = 678)	Targeted temperature management (n = 22)	Standard care (n = 656)	*p*-value
Age, y (SD^a^)	66.0 (16.1)	64.9 (17.0)	66.0 (16.1)	0.86
Male, n (%)	385 (56.8)	11 (50)	374 (57.0)	0.52
Comorbidities, n (%)				
Heart failure	158 (23.3)	6 (27.3)	152 (23.2)	0.61
Myocardial infarction	90 (13.3)	2 (9.1)	88 (13.4)	0.76
Arrhythmia	129 (19.0)	4 (18.2)	125 (19.1)	> 0.99
Hypotension before arrest	153 (22.6)	2 (9.1)	151 (23.0)	0.19
Respiratory insufficiency	480 (70.8)	10 (45.5)	470 (71.6)	0.01
Renal insufficiency	300 (44.2)	8 (36.4)	292 (44.5)	0.52
Regular dialysis	141 (20.8)	2 (9.1)	139 (21.2)	0.28
Hepatic insufficiency	134 (19.8)	2 (9.1)	132 (20.1)	0.28
Metabolic or electrolyte abnormality	134 (19.8)	6 (27.3)	128 (19.5)	0.41
Diabetes mellitus	238 (35.1)	8 (36.4)	230 (35.1)	> 0.99
Pneumonia	241 (35.5)	3 (13.6)	238 (36.3)	0.04
Bacteraemia	63 (9.3)	1 (4.5)	62 (9.5)	0.71
Metastatic cancer or any blood borne malignancy	156 (23.0)	0 (0)	156 (23.4)	0.004

^a^SD, standard deviation.

**Table 2:** Features, interventions, and outcomes of cardiac arrest events.

Variables	All patients (n = 678)	Targeted temperature management (n = 22)	Standard care (n = 656)	*p*-value
Arrest at night, n (%)	419 (61.8)	12 (54.5)	407 (62.0)	0.51
Arrest on weekend, n (%)	201 (29.6)	3 (13.6)	198 (30.2)	0.10
Arrest location, n (%)				<0.001
Intensive care unit	259 (38.2)	0 (0)	259 (39.5)	
General ward	349 (51.5)	6 (27.3)	343 (52.3)	
Emergency department	46 (6.8)	16 (72.7)	30 (4.6)	
Other locations	24 (3.5)	0 (0)	24 (3.7)	
Witnessed arrest, n (%)	441 (65.0)	15 (68.2)	426 (64.9)	0.82
Monitored status, n (%)	371 (54.8)	9 (40.9)	362 (55.3)	0.20
Shockable rhythm, n (%)	93 (13.7)	4 (18.2)	89 (13.6)	0.53
Critical care interventions in place at time of arrest, n (%)				
Mechanical ventilation	120 (17.7)	1 (4.5)	119 (18.1)	0.15
Antiarrhythmics	49 (7.2)	0 (0)	49 (7.5)	0.39
Vasopressors	245 (36.1)	1 (4.5)	244 (37.2)	0.001
Dialysis	52 (7.7)	1 (4.5)	51 (7.8)	> 0.99
Pulmonary artery catheter	7 (1.0)	0 (0)	7 (1.1)	> 0.99
Intra-aortic balloon pumping	7 (1.0)	0 (0)	7 (1.1)	> 0.99
CPR^a^ duration, min (SD^b^)	20.3 (19.2)	16.1 (10.6)	20.4 (19.4)	0.72
Body temperature during the first 24 hours after sustained ROSC^c^				
Fever,^d^ n (%)	173 (25.5)	3 (13.6)	170 (25.9)	0.32
Highest body temperature, °C (SD)	37.1 (1.6)	36.3 (1.3)	37.2 (1.6)	< 0.001
Lowest body temperature, °C (SD)	35.4 (1.4)	32.1 (1.0)	35.5 (1.3)	< 0.001
Percutaneous coronary intervention after sustained ROSC, n (%)	18 (2.7)	2 (9.1)	16 (2.4)	0.11
Withdrawal of life sustaining therapy	0 (0)	0 (0)	0 (0)	> 0.99
Survival to hospital discharge, n (%)	144 (21.2)	9 (40.9)	135 (20.6)	0.03
Favourable neurological outcome, n (%)	60 (8.8)	7 (31.8)	53 (8.8)	0.002

^a^CPR, cardiopulmonary resuscitation

^b^SD, standard deviation

^c^ROSC, return of spontaneous circulation

^d^Fever was defined as body temperature ≥ 38°C at least once during the first 24 hours after sustained ROSC.

**Table 3:** Reasons for potential exclusion from implementation of targeted temperature management of study patients.

Reasons for potential exclusion from targeted temperature management, n (%)	All patients (n = 678)	Targeted temperature management (n = 22)	Standard care (n = 656)	*p*-value
Pregnancy	2 (0.3)	0 (0)	2 (0.3)	> 0.99
Coagulopathy	161 (23.7)	8 (36.4)	153 (23.3)	0.20
Thrombocytopenia	140 (20.6)	3 (13.6)	137 (20.9)	0.59
Intracranial haemorrhage	21 (3.1)	0 (0)	21 (3.2)	> 0.99
Active bleeding	175 (25.8)	1 (4.5)	174 (26.5)	0.02
Acute stroke	31 (4.6)	0 (0)	31 (4.7)	0.62
No favourable neurological status within 24 hours before cardiac arrest	377 (55.6)	9 (40.9)	368 (56.1)	0.19
Hypotension after sustained ROSC^a^	152 (22.4)	1 (4.5)	151 (23.0)	0.04
With at least one of the above reasons	550 (81.1)	17 (77.3)	533 (81.3)	0.59

^a^ROSC, return of spontaneous circulation

**Table 4:** Multiple logistic regression models with clinical outcomes as the dependent variable. Goodness-of-fit assessment for favourable neurological outcome: n = 678, adjusted generalized *R*^2^ = 0.35, estimated area under the Receiver Operating Characteristic (ROC) curve = 0.86, and Hosmer and Lemeshow Chi-Squared test *p*-value = 0.87. Goodness-of-fit assessment for survival to hospital discharge: n = 678, adjusted generalized *R*^2^ = 0.2, estimated area under the Receiver Operating Characteristic (ROC) curve = 0.77, and Hosmer and Lemeshow Chi-Squared test *p*-value = 0.84.

Independent variables^a^	Odds ratio	95% confidence interval	*p-* value
*Dependent variable*: *Favourable neurological outcome*			
CPR^b^ duration	0.94	0.91–0.97	< 0.001
Shockable rhythm	3.35	1.64–6.75	< 0.001
Percutaneous coronary intervention after sustained ROSC^c^	6.42	2.08–20.54	0.001
Metastatic cancer or any blood borne malignancy	0.06	0.003–0.28	0.005
Age	0.97	0.96–0.99	0.006
Pneumonia	0.36	0.16–0.75	0.009
Pulmonary artery catheter in place at time of arrest	9.84	1.51–60.82	0.01
Male	2.35	1.20–4.88	0.02
** Targeted temperature management**	**3.74**	**1.19–11.00**	**0.02**
Renal insufficiency	0.50	0.26–0.93	0.03
*Dependent variable*: *Survival to hospital discharge*			
CPR duration	0.97	0.95–0.98	< 0.001
Metastatic cancer or any blood borne malignancy	0.30	0.16–0.57	< 0.001
Percutaneous coronary intervention after sustained ROSC	7.81	2.22–27.5	0.001
Hepatic insufficiency	0.34	0.18–0.66	0.002
Hypotension before arrest	0.40	0.23–0.71	0.002
Shockable rhythm	2.14	1.26–3.62	0.005
Renal insufficiency	0.52	0.31–0.86	0.01
Hypotension after sustained ROSC	0.49	0.28–0.86	0.01
Regular dialysis	2.05	1.14–3.69	0.02
** Targeted temperature management**	**1.41**	**0.54–3.66**	**0.48**

^a^The display of independent variables is arranged in order of *p*-values

^b^CPR, cardiopulmonary resuscitation

^c^ROSC, return of spontaneous circulation

All variables listed in Tables 1 to 3, except post-ROSC body temperature and percutaneous coronary intervention, were included in the variable selection procedure for the secondary outcome. As shown in Table 5, arrest in emergency department (OR: 22.48, 95% CI: 8.40–67.64; *p*-value < 0.001) was positively associated with TTM use and vasopressors in place at the time of arrest (OR: 0.08, 95% CI: 0.004–0.42; *p*-value = 0.02) was inversely associated with TTM use. Active bleeding (OR: 0.14, 95% CI: 0.02–1.08; *p*-value = 0.06) was marginally associated with a lower rate of TTM use.

**Table 5:** Multiple logistic regression models with implementation of targeted temperature management as the dependent variable. Goodness-of-fit assessment: n = 678, adjusted generalized *R*^2^ = 0.39, estimated area under the Receiver Operating Characteristic (ROC) curve = 0.88, and Hosmer-Lemeshow Chi-Squared test *p*-value > 0.99.

Independent variables^a^	Odds ratio	95% confidence interval	*p* value
Arrest in emergency department	22.48	8.40–67.64	< 0.001
Vasopressors in place at time of arrest	0.08	0.004–0.42	0.02
Active bleeding	0.14	0.02–1.08	0.06

^a^The display of independent variables is arranged in order of *p*-values

## Discussion

### Main Findings

TTM may benefit neurological recovery after IHCA but not survival. Laver et al. [13] reported that 50.9% of deaths following IHCA were caused by multiple organ failure and only 22.9% were caused by brain injury. Similarly, we found that poor prognostic factors for survival included hepatic failure, renal failure, and circulatory failure (hypotension before arrest) (Table 4). Therefore, for patients with these factors, TTM might not be as beneficial as it is for OHCA patients [4,5]. In contrast, for those who are likely to survive IHCA, TTM might exert numerous beneficial effects that improve the chances for neurological recovery.^12^ Further, our analysis also revealed that the effects of TTM were independent of the influence of initial arrest rhythms, which supported the recommendations of resuscitation guidelines [10,11], i.e., TTM should be used in all IHCA patients with all rhythm types.

### Comparison with Previous Studies on TTM for IHCA

Few studies have been dedicated to the investigation of TTM use for IHCA. In a small before-and-after study, Kory et al. [14] reported that TTM did not benefit the neurological outcomes of IHCA. Later, Nichol et al. [15] examined a nationwide database and revealed that neither neurological nor survival outcomes were improved by TTM in IHCA occurring in general wards. However, Kory et al. [14] did not account for the difference in baseline patient characteristics in the statistical analysis, which might bias the results. And, although Nichol et al. [15] used advanced statistical analysis to adjust for baseline differences, the significant misclassification bias caused by the data collection method may nullify the associations between TTM and outcomes. For example, in the TTM group, nearly half (48%) of patients who were claimed to have received TTM had no documented body temperature below 34°C or no recorded body temperature at all [15].

In a recent study, Perman et al. [17] investigated TTM use in PCAS with non-shockable rhythms. The subgroup analysis demonstrated that TTM may benefit neurological outcomes in IHCA [17]. However, although the included patients were eligible for TTM, the distribution of reasons for potential exclusion from TTM was not revealed. In the TTM trial, Nielsen et al. [8] reported that 1242 patients were eligible for TTM after initial screening, but 292 patients (23.5%) were excluded because of presence of at least one reason for potential exclusion. Therefore, even if Perman et al. [17] had used propensity scores to match patients receiving TTM and those receiving standard care, a question would still remain whether the patients receiving standard care would have not been considered for TTM from the beginning because of the presence of reasons for exclusion.

Also, since these studies [14,15,17] did not reveal the temperature profiles of included patients, it is difficult to determine whether the claimed benefit of TTM was simply caused by avoidance of fever in the TTM group, since fever is a known risk factor for post-ROSC neurological disability [22]. In fact, these studies included control patients from before 2005; it was not until after this time that the importance of temperature management was emphasized in resuscitation guidelines [6]. In comparison, the temperature profiles or incidence of fever was reported in our study clearly.

### Features of Current Analysis

First, we did not use propensity scores in our analysis, as previous studies did [15,17]. Propensity score analysis is useful if the treatment is common and the outcome is rare [23,24]. Only 3.2% (22/678) of patients included in our study received TTM. If propensity score matching was used, a large proportion of patients from both intervention and control groups would be excluded because of a lack of matches. This may significantly alter the composition of the study population [23,24]. Therefore, in our situation, propensity score analysis may not be a better choice than multivariable logistic regression [23,24]. The most important step toward a less biased result is whether the considerations for a specific treatment, including indications and reasons for potential exclusion from its use, were comprehensive and included in the analysis [23,24].

Second, we explicitly stated the policy of WLST in our hospital. Mulder et al. [25] noted that 81% of patients who died after OHCA did so after WLST. Albaeni et al. [26] indicated that for more than 50% of OHCA patients, care was often withdrawn prematurely, before the accepted time for neurological recovery (i.e., 72 hours after ROSC) [7]. It has been reported that approximately 32% of comatose survivors of OHCA treated with TTM woke with favourable neurological outcome after 72 hours [25]. As a result, if the policy of WLST was not considered in the analysis, it might be difficult to refute whether the observed benefits of TTM were caused by its comparison with the control group for whom the WLST was prematurely determined. In our investigation, no patients experienced WLST because of poor neuroprognosis. The extremely low rate of WLST in our cohort was expected because WLST was not yet allowed by legislation during the study period. Our results are also consistent with reports from other areas of Asia [27].

Finally, we excluded patients receiving ECPR because the protocols for TTM were different between patients with and without ECPR. Also, ECPR was often used as a final, desperate effort with prolonged CPR duration [28], which may make patients receiving ECPR distinct from patients who did not receive ECPR. Therefore, the exclusion of patients receiving ECPR from our analysis may be reasonable.

### Factors Influencing TTM Use for IHCA

Mikkelsen et al. [16] reported that TTM was initiated in approximately 2% (1367/ 67,498) of IHCA patients, which was similar to the rate observed in our investigation (3.2%; 22/678). Mikkelsen et al. [16] also indicated that patient age, time and location of arrest, and type of hospital were associated with TTM use. Mikkelsen et al. [16] concluded that the appropriateness of the use of TTM in the available dataset could not be determined. Therefore, it is still unclear whether the prevalence of reasons for potential exclusion affects the use of TTM for IHCA.

Our investigation revealed that 81.1% (550/678) of comatose survivors of IHCA had at least one reason for potential exclusion from TTM use. The exclusion criteria used in our hospital were generally consistent with those proposed in previous OHCA trials [4,5,8]. However, the proportion of patients with reasons for potential exclusion was higher than that reported by Nielsen et al. [8], who excluded 23.5% (292/1242) patients in the TTM trial because of similar reasons. If the exclusion criteria were strictly executed in our clinical practice as in a clinical trial, the number of IHCA patients suitable for TTM would only be 128 (678–550), which equates to 8.3% (128/1540) of the total number of IHCA patients in our hospital in one decade. Interestingly, our results suggest that even if patients have one reason for potential exclusion, clinicians may still attempt to use TTM. Only the presence of active bleeding was shown to marginally discourage clinicians from TTM use for IHCA. Notably, none of the proposed exclusion criteria were associated with neurological outcome. Therefore, if a trial of TTM is planned for IHCA, exclusion criteria should be refined; otherwise, the enrolment process may be prolonged.

Arrest in the emergency department was significantly correlated with increased TTM use, which was similar to the findings by Mikkelsen et al. [16]. In our hospital, the most experienced and well-trained staff in TTM were at the intensive care unit of emergency department because of their familiarity with OHCA treatment. Although features of IHCA that occurs in the emergency department might be different from those that occur in a general ward or intensive care unit, the team in charge of the post-resuscitation care might be the main determinant of TTM use for IHCA in our hospital. Therefore, the results of our analysis may still be generalizable to other IHCA populations and not restricted to a certain area within the hospital.

### Application in Clinical Practice

The latest resuscitation guidelines [10,11] differ on the recommendation level of TTM use for IHCA: the AHA [10] promotes a stronger recommendation than the ERC [11]. Although a clinical trial is considered to provide the best evidence, difficulties in enrolling patients may prolong the completion of trials. Despite the fact that our study was retrospective in design, we applied cautious analyses and thoughtful considerations of multiple confounders. As such, our results might serve as evidence of the effectiveness of TTM for IHCA. Furthermore, studies have indicated that TTM may not increase the rate of complications feared by most clinicians, including haemorrhage [29] or infection [30]. Therefore, TTM should still be considered an effective strategy to improve the usually bleak prognosis of IHCA and its use should be encouraged for appropriate patients experiencing IHCA [31].

### Study Limitations

Several limitations of our study should be considered. First, it is possible that patients receiving TTM demanded more resources, including experienced nursing staff, and, therefore, the outcomes after TTM were better than those in patients who did not receive TTM. Also, TTM usually involves a well-designed bundle of care, which might also explain the observed benefit of TTM. Nonetheless, even if patients did not receive TTM, the standard level of care may not differ significantly from TTM in our hospital, which is a tertiary medical centre. Second, we only recorded the neurological outcome at hospital discharge and did not conduct long-term follow-up. Still, neurological status at hospital discharge correlates with long-term outcomes [32]. TTM may benefit long-term outcomes, but a prospective study with long-term observation is needed to confirm this hypothesis.

## Conclusions

TTM might be associated with favourable neurological outcome of IHCA patients, irrespective of arrest rhythms. Although the prevalence of proposed exclusion criteria for TTM was high among IHCA patients, these factors did not influence the decision of TTM use in clinical practice or neurological outcomes after IHCA.

## Supporting Information

S1 Dataset Raw data used in statistical analysis. (XLSX)
